# Genomic features of three major diarrhoeagenic Escherichia coli pathotypes in India

**DOI:** 10.1099/mgen.0.001430

**Published:** 2025-07-07

**Authors:** Yuki Hoshiko, Goutam Chowdhury, Kei Kitahara, Debjani Ghosh, Debora Satie Nagano, Ayumu Ohno, Shin-ichi Miyoshi, Miki Okuno, Takeshi Yamamoto, Shanta Dutta, Asish K. Mukhopadhyay, Yoshitoshi Ogura

**Affiliations:** 1 Division of Microbiology, Department of Infectious Medicine, Kurume University School of Medicine, Kurume, Fukuoka, Japan; 2 Laboratory of Environmental Hygiene, Department of Health Science, School of Allied Health Sciences, Kitasato University, Kanagawa, Japan; 3 Division of Bacteriology, ICMR-National Institute of Cholera and Enteric Diseases, Kolkata, India; 4 Collaborative Research Centre of Okayama University for Infectious Diseases, ICMR-National Institute of Cholera and Enteric Diseases, Kolkata, India; 5 Graduate School of Medicine, Dentistry and Pharmaceutical Sciences, Okayama University, Okayama, Japan; 6 Research Centre for Intestinal Health Science, Okayama University, Okayama, Japan

**Keywords:** antimicrobial resistance, diarrhoeagenic *Escherichia coli*, genome, India, virulence gene

## Abstract

**Background.** Diarrhoea remains a major threat to children in developing nations, with diarrhoeagenic *Escherichia coli* (DEC) being the primary causative agent. Characterizing prevalent DEC strains is crucial, yet comprehensive genomic analyses of major DEC strains, including enteropathogenic *E. coli* (EPEC), enteroaggregative *E. coli* (EAEC) and enterotoxigenic *E. coli* (ETEC), are lacking in India.

**Methods.** We sequenced 24 EAEC and 23 EPEC strains from Indian patients with diarrhoea and conducted an extensive database search for DEC human isolates from India. Detailed phylogenetic analyses, virulence gene subtyping and examinations of accessory virulence and antimicrobial resistance (AMR) genes were performed.

**Results.** The analysed DEC strains included 32 EAEC, 25 EPEC, 32 ETEC and 1 each of the EPEC/ETEC-hybrid and ETEC/EAEC-hybrid pathotypes. These strains were predominantly classified into phylogroups A (35.2%) and B1 (41.8%) and dispersed within these phylogroups without pathotype-specific clustering. One ETEC strain was classified into cryptic clade 1. Subtypes of hallmark virulence genes varied substantially amongst strains in each pathotype, and 31 accessory virulence genes were detected either specifically within certain pathotypes or across multiple pathotypes at varying frequencies, indicating diversification of the virulence gene repertoire within each pathotype. Acquired AMR genes were found in 73.6% of the strains, with frequent identification of AMR genes for aminoglycosides (40.0%), *β*-lactams (64.8%), sulphonamides (49.5%) and trimethoprim (42.9%). Known quinolone-resistant mutations were found in 74.7% of the strains, whereas AMR genes for macrolide (30.8%), phenicol (11.0%) and tetracycline (27.4%) were less frequent.

**Conclusions.** The diverse virulence potential and trends in AMR gene prevalence amongst major DEC strains in India are highlighted in this study. Continuous monitoring of DEC strain characteristics is essential for the effective control and treatment of DEC infections in India.

**Impact Statement**

In developing countries, diarrhoea remains a significant cause of childhood mortality, with diarrhoeagenic *E. coli* (DEC) being one of the primary causative agents. DEC strains are categorized into at least five pathotypes, each of which possesses distinct virulence factors and causes different symptoms. The high prevalence of antimicrobial-resistant DEC strains also poses a significant burden. Information on the genetic traits of virulence and antimicrobial resistance in prevalent DEC strains in these regions is limited. In this study, we conducted a comprehensive genomic analysis of the major DEC pathotypes in India to elucidate their genetic characteristics. Each pathotype exhibited significant diversity in the number and repertoire of virulence genes, indicating varying levels of pathogenicity. We also identified infections caused by strains exhibiting hybrid pathotypes and the newly recognized emerging pathogen cryptic clade 1. Antimicrobial resistance was notably advanced, particularly against *β*-lactams, aminoglycosides, sulphonamides, trimethoprims and quinolones, although resistance to phenicols, macrolides and tetracyclines was relatively low. Continuous genetic surveillance of DEC strains is crucial for the effective control and treatment of DEC infections.

## Data Summary

All sequencing data generated in this study have been deposited in DDBJ/ENA/GenBank under the BioProject accession number PRJDB18117. The list of Indian isolates from public databases can be accessed through the Supplementary data files.

## Introduction

Diarrhoea poses a great threat to children younger than 5 years of age in South Asia and sub-Saharan Africa [1]. Amongst the various pathogens belonging to viruses, bacteria, protozoa and parasites, diarrhoeagenic *Escherichia coli* (DEC) is one of the most common causes of diarrhoea in low- and middle-income countries [2]. In India, 30% of diarrhoea is attributed to DEC [3]; thus, emphasizing the genetic features of prevalent DEC strains is important for the effective prevention and control of diarrhoeal infection.

DEC is primarily classified into five distinct pathotypes, namely, Shiga toxin (Stx)-producing *E. coli* (STEC), enterotoxigenic *E. coli* (ETEC), enteropathogenic *E. coli* (EPEC), enteroaggregative *E. coli* (EAEC) and enteroinvasive *E. coli*, each of which causes diverse clinical manifestations by means of individually acquired virulence determinants [4]. Although STEC is the foremost concern amongst DEC strains in developed nations such as Japan, the USA and Europe [4], the predominant pathotypes responsible for diarrhoea in India include ETEC, EPEC and EAEC [5]. The low prevalence of STEC infections in India is not fully understood, but one possible reason is that a large portion of the population avoids beef, the primary source of STEC infection, due to religious beliefs [6].

ETEC refers to strains of *E. coli* that produce at least one type of two defined groups of enterotoxins: heat-labile enterotoxin (LT) and heat-stable enterotoxin (ST) [4]. STs are subdivided into STa, which is primarily associated with human disease, and STb, which is predominantly found in ETEC strains of swine origin [7]. Similarly, LT is classified into two antigenically distinct groups: LT-I, found in both human- and swine-derived ETEC strains, and LT-II, which has been isolated from humans and a range of other hosts [7].

EPEC are *E. coli* strains that cause diarrhoea and distinct attaching and effacing (A/E) lesions on the intestinal epithelium [7]. They lack Stx and LT or ST enterotoxins. EPEC strains carry the locus of enterocyte effacement (LEE), which encodes a type 3 secretion system and seven secretion effectors, facilitating the formation of A/E lesions and promoting intimate adherence to epithelial cells [7]. In addition to LEE-encoded effectors, more than 30 effectors have been identified on other mobile genomic elements, notably prophages, and these are designated non-LEE effectors [8]. These non-LEE effectors are hypothesized to play a pivotal role in human pathogenesis [9]. EPEC strains have been classified into typical EPEC (tEPEC), which possess a large virulence plasmid encoding type IV fimbriae known as the bundle-forming pilus (BFP), and atypical EPEC (aEPEC), which lack this plasmid [4].

EAEC, which is defined as *E. coli* exhibiting characteristic aggregative adherence to HEp-2 epithelial cells whilst lacking the primary genetic markers of other DEC pathotypes, comprises genetically diverse strains harbouring a heterogeneous array of virulence genes [7]. Most EAEC strains harbour the plasmid-encoded transcriptional activator AggR, which controls the expression of various virulence factors, including aggregative adherence fimbriae (AAF). A study of both symptomatic and asymptomatic carriers revealed that the carriage of the AggR regulon exhibited a significantly greater correlation with symptoms, leading to the designation of AggR-positive strains as typical EAEC strains [10]. The plasmid-encoded *aatA* gene and the chromosomally encoded *aaiC* gene are frequently employed as molecular markers for the identification of EAEC [11,12].

In addition, various accessory virulence factors have been identified in each pathotype [13,14]. Although the roles of most accessory virulence factors in infection and pathogenesis have not yet been fully elucidated, their repertoire is believed to be crucial for the development of full virulence in DEC [15,16].

In diarrhoea-endemic regions, antimicrobial resistance (AMR) of DECs is also a major public health concern [17]. Although antimicrobial therapy for DEC infections is effective in managing severe diarrhoeal episodes in high-risk patients and reducing the duration of symptoms in cases of traveller’s diarrhoea, excessive antimicrobial use can lead to the emergence and dissemination of AMR strains [18,19]. In Kolkata, India, more than half of DEC strains have been reported to exhibit multidrug resistance, defined as resistance to more than three antimicrobial classes, with ~10% displaying resistance to six different classes [5]. To control AMR strains, elucidating the underlying genetics of AMR and using this knowledge as the basis for extensive surveillance of AMR strains are paramount. Nevertheless, there has not been a comprehensive genomic investigation of DEC to unveil the genomic attributes related to virulence and AMR in India.

In this study, we sequenced the genomes of 24 EAEC and 23 EPEC strains isolated from 2019 to 2023 in Kolkata, India. Furthermore, we conducted a public database search and retrieved genome data for 8 EAEC strains, 2 EPEC strains and 32 ETEC strains, as well as one strain each of EPEC/ETEC-hybrid and ETEC/EAEC-hybrid pathotypes. Using this genomic information, we identified virulence genes and AMR genes and evaluated their prevalence in India.

## Methods

### Isolation of DEC strains

In this study, stool specimens (*n*=1,853) were tested from acute diarrhoeal patients treated at the Infectious Diseases and Beleghata General Hospital and Dr. B. C. Roy Post-graduate Institute of Pediatric Sciences in Kolkata, India, from 2019 to 2023. For the isolation of major DEC strains, a loopful of stool sample was streaked on MacConkey agar (Difco, BD, USA) plates, followed by incubation for 16–18 h at 37 °C. Three typical lactose-fermenting pink colonies from each sample were selected and subcultured on Luria agar (LA) (Difco, BD, USA) plates. For the presumptive identification of *E. coli*, cultures from the Luria agar (LA) plate were checked for oxidase negativity, acid/gas production in triple sugar iron agar and positive indole production [12]. A small portion of indole-positive bacteria was taken from each of the three colonies plated on LA plates and mixed with 500 µl of PBS in 1.5 ml microfuge tubes and boiled for 10 min in a water bath followed by snap chilling on ice for 5 min. The heat-treated bacterial suspensions were centrifuged at 10,000 r.p.m. for 5 min to pellet down the cell debris, and the supernatants were used as DNA templates for PCR. Confirmation was performed using multiplex PCR for the detection of virulence marker genes for each pathotype. Multiplex PCR was carried out to assess the presence of specific virulence genetic markers: *est* encoding ST and/or *elt* encoding LT for ETEC, *eae* encoding intimin and/or *bfpA* encoding BFP for EPEC and *aatA* encoding dispersin and/or *aaiC* encoding a secretion system for EAEC. A total of six primer pairs were used to analyse different virulence-associated genes. PCR was performed according to published procedures [12]. The presence of at least one of the virulence genes was considered positive for the respective DEC pathotype [12]. The *E. coli* isolates were also screened by PCR for the presence of Stx genes (*stx1* and *stx2*) and the *ipaH* gene. However, none of the isolates tested positive for these genes.

### Genome sequencing, assembly and annotation

Genomic DNA from the DEC strains isolated in this study was purified using a QIAamp DNA Mini Kit (QIAGEN, Hilden, Germany). Subsequently, libraries were prepared using the xGen DNA Library Prep EZ Kit (Integrated DNA Technologies), in conjunction with NEBNext Multiplex Oligos for Illumina (96 Unique Dual Index Primer Pairs) (New England BioLabs). Sequencing was conducted on the Illumina NovaSeq X plus platform to generate paired-end reads of 151 bp. Genome assembly of the Illumina sequence reads was performed using Platanus_B v1.3.2 with the default settings [20]. The assessment of sequence quality was based on the criteria of achieving 99% or greater completeness and less than 3% contamination, employing CheckM v1.2.0 software [21]. Gene identification and annotation procedures were carried out using Prokka v1.14.6 with the default settings [22]. All sequence data generated in this study have been submitted to the NCBI BioProject database under accession number PRJDB18117.

### Genome data search in the public database

We initially obtained 118 assembled genomes, encompassing all the genomes of *E. coli* strains isolated from human stool in India available in EnteroBase [23] (last accessed: 07 February 2024). All the data passed quality checks (>95% completeness and <5% contamination) using CheckM to eliminate low-quality assemblies. Subsequently, blastn searches of the marker genes for EPEC, ETEC and EAEC were conducted against the obtained genomes to identify the three DEC pathotypes. Ultimately, the genomes of 44 isolates were selected for further analysis, and the strains are listed in Table S1, available in the online Supplementary Material. The remaining non-DEC strains (*n*=60) were used as controls and are listed in Table S2.

### *In silico* serotyping and sequence typing

*In silico* serotyping was conducted by using ECTyper version v1.0.0 with the default settings. The sequence types (STs) of the strains analysed in this study, as well as those retrieved from public databases, were identified using SRST2 and PubMLST [24] based on MLST of internal fragments of seven housekeeping genes: *adk*, *fumC*, *gyrB*, *icd*, *mdh*, *purA* and *recA*.

### Identification of virulence and AMR genes

The identification and subtyping of *elt*, *est* and *eae* for each DEC pathotype were carried out using blastn searches against a comprehensive database encompassing all known subtypes of these virulence genes, as described by Okuno *et al*. [25], applying thresholds of 99% sequence identity and 99% coverage. Genes for non-LEE effectors were identified through a tblastn search using a database previously described by Arimizu *et al*., with thresholds of 50% identity and 50% coverage. The identification of known ETEC colonization factors (CFs) was performed via a blastn search against the database file ETEC_vir_db (https://github.com/avonm/ETEC_vir_db). A result was considered positive when all genes of a given CF were conserved (>90% identity and >80% coverage). The identification of the other known *E. coli* virulence genes was carried out via blastn searches with the database described by Arimizu *et al*. [26] and the Virulence Factor Database [13], employing thresholds of 50% identity and 50% coverage. In all blast searches, when multiple genes exceeded the threshold, the top hit was selected.

The acquired AMR genes and plasmid replicon sequences were identified using ABRicate 1.0.1 (https://github.com/tseemann/abricate) with the default settings, employing the ARG-ANNOT [27] and PlasmidFinder [28] databases, respectively. Additionally, mutations within the quinolone resistance-determining regions (QRDRs) of *gyrA*, *gyrB*, *parC* and *parE* were analysed using the NCBI Antimicrobial Resistance Gene Finder (AMRFinderPlus) version 3.11.14 with default settings [29].

### Core gene-based phylogenetic analysis

To construct a core gene-based phylogenetic tree, pan-genomic analysis was conducted using Roary v3.13.3 [30], with a 90% sequence identity threshold. SNP sites were extracted from the core gene alignment using snp-sites v2.5.1 with the -c option [31]. Subsequently, a maximum likelihood (ML) phylogenomic tree was constructed using RAxML-NG ver. 1.0.1 [32] with the following parameters: --all, --bs-trees 100 and --model GTR+G4. The ML tree was visualized and annotated using iTOL v6.6 [33].

### Co-occurrence network analysis

To investigate the co-occurrence patterns of AMR genes and plasmid replicons amongst the 91 *E. coli* strains, gene presence/absence data obtained from ABRicate were utilized to construct pairwise co-occurrence matrices for both AMR genes and plasmid replicons. Hierarchical clustering of gene communities and network visualization were conducted using the igraph package v2.1.2 [34] in R v4.4.2 (R Core Team, 2021).

## Results

### DEC strains

In this study, we sequenced the genomes of 24 EAEC strains and 23 EPEC strains isolated from paediatric patients under 5 years of age suffering from diarrhoea who sought medical attention in hospitals located in Kolkata between 2019 and 2023. Other pathogens, including rotavirus and *Aeromonas* species, were also concurrently detected in some patients (Table S1). We then undertook a comprehensive exploration of the genomic repository for DEC isolates originating from India. Amongst the publicly available *E. coli* genomic datasets, 118 strains were sourced from human subjects in India. By identifying the marker genes associated with each pathotype of DEC, we acquired sequencing data for 8 EAEC strains, 2 EPEC strains, 32 ETEC strains and 1 EPEC/ETEC-hybrid strain from the database. Additionally, one ETEC strain, positive for both *estA* and *aggR* genes but lacking *aatA* and *aaiC*, was included as an ETEC/EAEC-hybrid strain from the database (Table S1). Additionally, 60 stool isolates lacking DEC marker genes were obtained from public databases and included as non-DEC isolates for comparison.

### Hallmark virulence genes

Five types of AAFs, which serve as the hallmark virulence factors for EAEC, have been identified. Amongst the 32 EAEC strains analysed, four types of AAF genes [*aagA* (AAF-I), *aafA* (AAF-II), *agg3A* (AAF-III) and *aaf5A* (AAF-V)] were identified in 16 strains, with seven strains having *aaf5A* being the most common (Table 1). None of the AAF types were detected in the remaining 16 strains. Instead, two of the AAF-negative strains were positive for the CF CS22 (Table S1). Amongst the 32 ETEC strains analysed, 20 strains had *esta* alone, 1 strain had *elt1* alone and 11 strains co-harboured both *estb* and *elt1* simultaneously. One to three CFs were identified in 30 ETEC strains, with CS3 being the most prevalent (*n*=8), followed by CS6 (*n*=7), CFA/I (*n*=6) and CS21 (*n*=6). No known CFs were identified in the remaining two strains. The intimin gene (*eae*), a marker gene for EPEC, has been classified into 30 subtypes [35]. The *eae* genes of the 25 EPEC strains analysed were classified into 13 subtypes, with a dominance of seven strains possessing *eae*_β1. *bfpA*, a gene of the BFP operon, was identified in two of the EPEC strains analysed (Table S1).

**Table 1.** Subtype of the hallmark virulence genes in each pathotype of DEC

Pathotype	Virulence gene subtype	No. of strain
**EAEC**	*aggA*	4
	*aafA*	1
	*agg3A*	4
	*aaf5A*	7
	Unclassified	16
	Total	32
**EPEC**	*eaeA*_alpha1	1
	*eaeA*_alpha2	1
	*eaeA*_beta1	7
	*eaeA*_beta2	2
	*eaeA*_epsilon2	2
	*eaeA*_eta2	3
	*eaeA*_gamma1	1
	*eaeA*_kappa	2
	*eaeA*_lambda	1
	*eaeA*_mu	1
	*eaeA*_rho	1
	*eaeA*_theta	2
	*eaeA*_zeta	1
	total	25
**ETEC**	*esta*	20
	*elt1*	1
	*esta, elt1*	11
	total	32

### Phylogenetic features

**Fig. 1.**
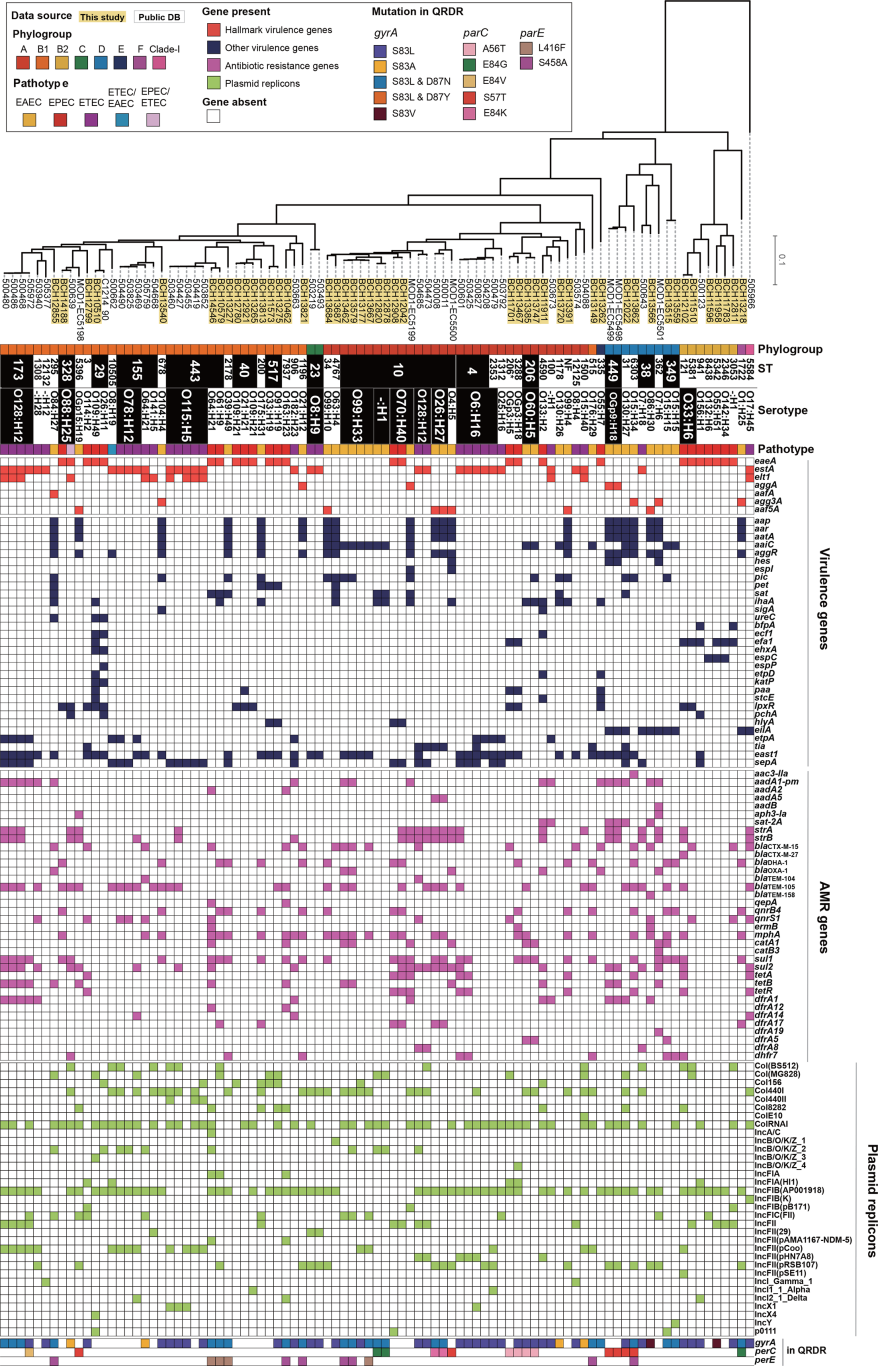
Phylogenomic relationships of Indian DEC strains and the distribution of virulence genes, AMR genes and plasmid replicons in these strains. The ML tree of 91 isolates from ETEC, EPEC and EAEC was constructed based on 212,286 SNP sites located on 2,917 core genes. Information about the phylogroup, ST and serotype and the distribution of virulence genes, acquired AMR genes, plasmid replicons and QRDR mutations for each strain is also provided.

In both pathotypes, strains were predominantly classified into phylogroups A (35.2%) and B1 (41.8%), with a subset classified as B2 in EPEC and D in EAEC (Table 2). One strain designated ETEC was assigned to cryptic clade 1. Despite the identification of several closely related clonal lineages (Fig. S1), strains belonging to ETEC, EPEC and EAEC exhibited variable distributions within a core-gene-based ML tree (Fig. 1). The DEC strains were classified into 51 distinct STs, including a single-locus variant of ST206 (*n*=1) and a non-typeable variant (*n*=1). Fourteen STs included multiple strains, ranging from 2 to 14 strains, whilst the remaining STs consisted of a single strain. Amongst these STs, ST10 and ST443 were the predominant STs, and ST10, ST38, ST328 and ST443 were shared amongst the different pathotypes of DEC strains. Although six strains were O-untypeable, the remaining strains were distributed across 56 different serotypes. O115:H5 and O6:H16, each comprising five strains, were the most prevalent serotypes. O70:H40 and O88:H25 were shared amongst the different pathotypes of the strains.

**Table 2.** Phylotypes of DEC isolates

Pathotype	No. of strains in each phylogroup (%)								
	A	B1	B2	C	D	E	F	Clade I	Total
EAEC	16 (50.0)	7 (21.9)	0	0	8 (25.0)	0	1 (3.1)	0	32 (100)
EPEC	5 (20.0)	12 (48.0)	7 (28.0)	0	0	1 (4.0)	0	0	25 (100)
ETEC	11 (34.4)	17 (53.1)	0	2 (6.3)	1 (3.1)	0	0	1 (3.1)	32 (100)
EPEC/ETEC hybrid	0	1 (100)	0	0	0	0	0	0	1 (100)
ETEC/EAEC hybrid	0	1 (100)	0	0	0	0	0	0	1 (100)
Non-DEC	23 (38.3)	20 (33.3)	6 (10.0)	1 (1.7)	0	0	9 (15.0)	1 (1.7)	60 (100)
Total	32 (35.2)	38 (41.8)	7 (7.7)	2 (2.2)	9 (9.9)	1 (1.2)	1 (1.1)	1 (1.1)	91 (100)

**Fig. 2.**
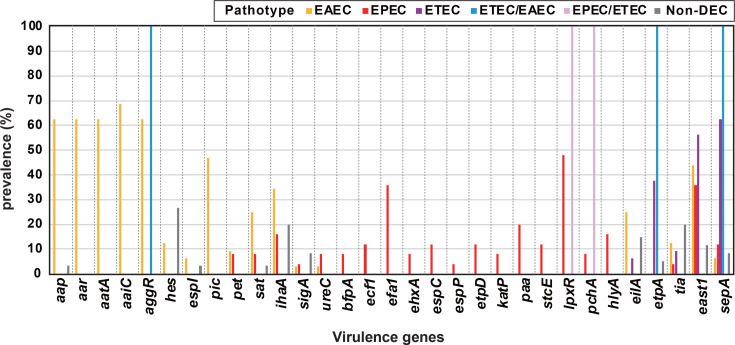
Distribution of accessory virulence genes in the DEC strains. The distribution of 31 accessory virulence genes across each DEC pathotype and non-DEC isolate is illustrated. Gene presence or absence was determined through blastn homology analysis, employing thresholds of 50% sequence identity and 50% coverage.

### Accessory virulence genes

We investigated the distribution of other known *E. coli* virulence genes amongst the DEC strains analysed in this study. Combinations of 31 virulence genes, ranging from 1 to 11 genes per strain, were identified amongst 87 strains. Each gene was detected in between 1 and 41 strains (Figs.1  2 and Table S1). A phylogenetically biassed distribution was not observed for any of the virulence genes (Fig. 1). Conversely, many virulence genes were detected in a pathotype-specific manner (Fig. 2). Seven genes were specifically identified in the EAEC strains: *aap*, *aar*, *aatA*, *aaiC*, *aggR*, *hes*, *espI* and *pic*, although *aggR* was also present in an ETEC/EAEC-hybrid strain. *aggR* was identified in 20 of the EAEC strains, and *aap*, *aar* and *aatA* were identified in all *aggR*-positive strains, whereas *aaiC* was in only half of them (Table S1). Five genes, *pet*, *sat*, *ihaA*, *sigA* and *ureC*, were identified in multiple EAEC and EPEC strains, whilst *eilA* was detected in several EAEC and ETEC strains (Fig. 2). Ten genes (*bfpA*, *ecf1*, *efa1*, *ehxA*, *espC*, *espP*, *etpD*, *katP*, *paa* and *stcE*) were specifically detected in EPEC strains, albeit with a frequency of less than 50%. Three genes, *IpxR*, *pchA* and *hylA*, were detected in both the EPEC and EPEC/ETEC-hybrid pathotypes. Five genes were identified in the ETEC strains, none of which were exclusive to this pathotype. *etpA* and *sepA* were identified in both ETEC and ETEC/EAEC-hybrid pathotypes. *tia*, *east1* and *sepA* were detected across three pathotypes: EAEC, EPEC and ETEC. In non-DEC stool isolates, *hes*, *ihaA*, *eilA* and *tia* were identified relatively frequently amongst the accessory virulence genes (Fig. 2).

**Fig. 3.**
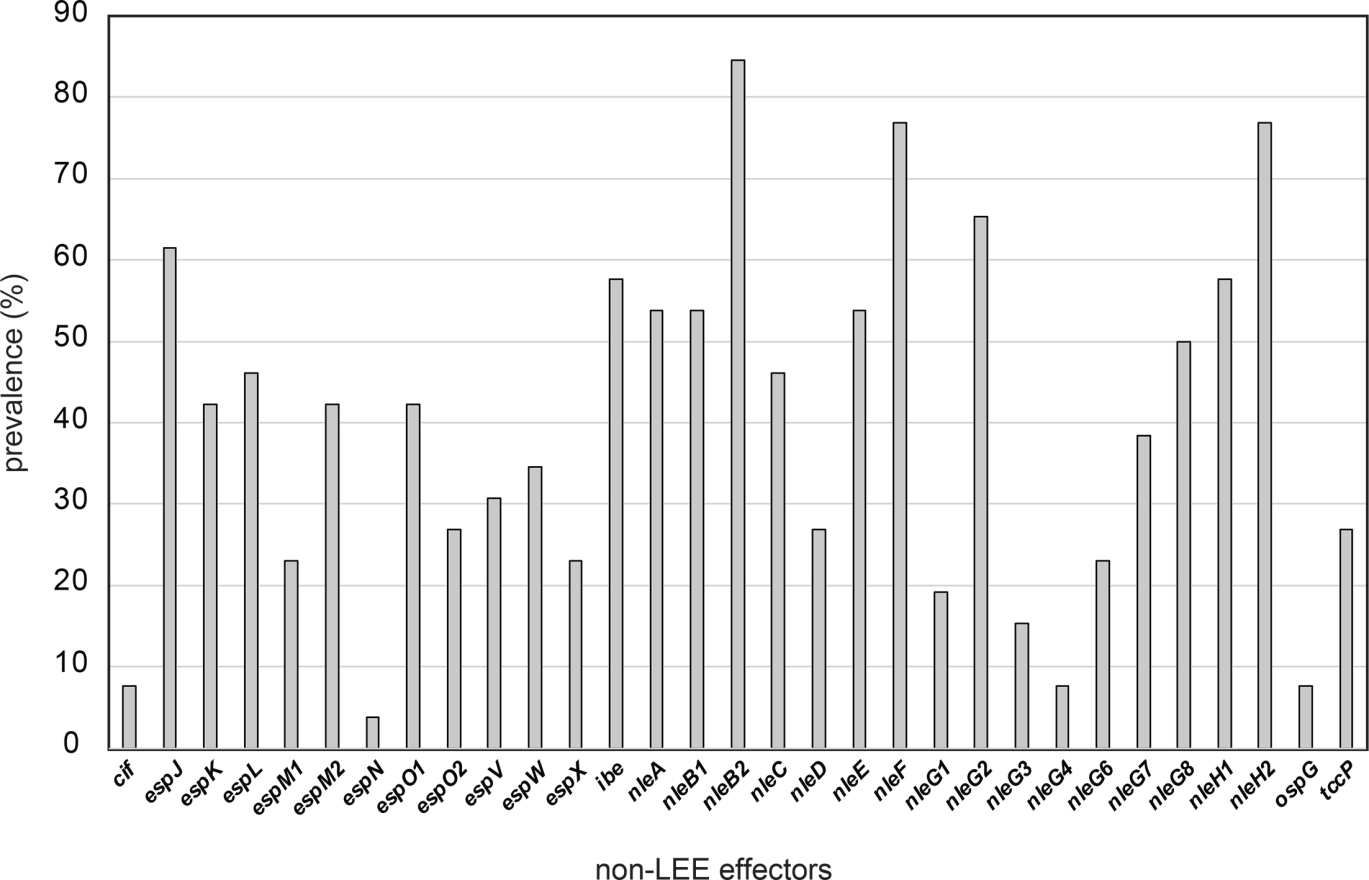
Distribution of non-LEE effectors in the EPEC strains. The prevalence of non-LEE-encoded effectors in the EPEC strains is depicted. Gene presence or absence was determined through blastn homology analysis, employing thresholds of 50% sequence identity and 50% coverage.

A total of 33 non-LEE effectors were identified in the EPEC and EPEC/ETEC-hybrid strains, whilst none of these effectors were identified in the other pathotypes (Fig. 3 and Tables S1 and S3). The conservation levels of the non-LEE effectors exhibited a considerable range, spanning from 3.8% to 84.6%. Within each strain, a variable number of effectors, ranging from 2 to 25, were detected. No significant differences in the non-LEE effector carriage status of the strains were identified with respect to ST, phylogroup, serotype and year of isolation, potentially owing to the limited sample size (Table S1).

### AMR genes and mutations

**Fig. 4.**
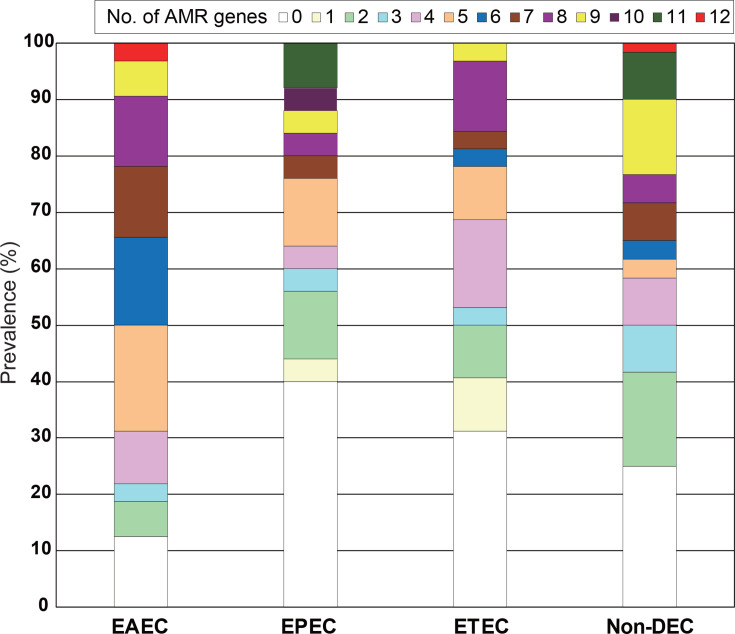
Prevalence of strains carrying AMR genes amongst the EAEC, EPEC and ETEC strains. Bar graphs indicate the percentage of strains carrying different numbers of AMR genes in each pathotype of strains. The acquired AMR genes were identified using ABRicate with the default settings and the ARG-ANNOT database.

One or more AMR genes (up to 12 genes) spanning eight distinct antimicrobial classes were identified in 73.6% of the DEC strains (Fig. 4 and Table S1). The strains harbouring AMR genes exhibited a dispersed distribution across the phylogeny of the DEC strains (Fig. 1). More than 30% of the EPEC and ETEC strains were negative for known AMR genes, whilst one or more AMR genes were detected in 87.5% of the EAEC strains (Fig. 4). Amongst non-DEC strains, 75% carried one or more AMR genes, a proportion similar to that observed in the overall DEC strains.

**Fig. 5.**
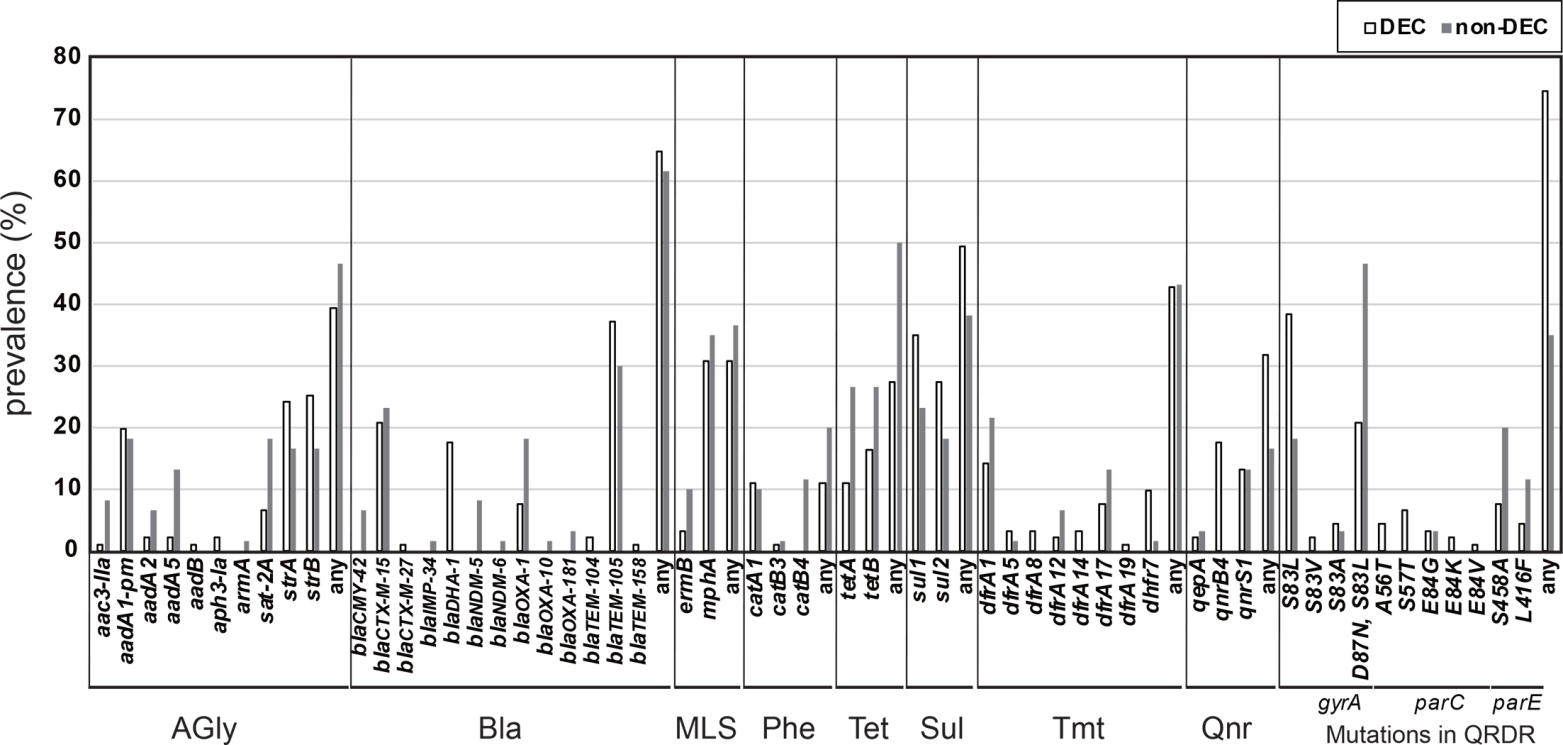
Distribution of acquired AMR genes and mutations in the QRDRs of DEC strains. Bar graphs show the prevalence of each resistance gene in the DEC and non-DEC isolates. The acquired AMR genes were identified using ABRicate with the default settings and the ARG-ANNOT database. Mutations within the QRDRs of *gyrA*, *gyrB*, *parC* and *parE* were analysed using the NCBI Antimicrobial Resistance Gene Finder with default settings. Only mutations with experimentally confirmed quinolone resistance are shown.

Forty percent of strains were positive for one or more aminoglycoside resistance genes, with *strB* being the most frequently detected (Fig. 5). *β*-Lactamase genes were identified in 67% of the DEC strains, with the most commonly detected *β*-lactamase gene being *bla*_TEM-105_. Expanded-spectrum *β*-lactamase (ESBL) genes, namely, *bla*_CTX-M15_, *bla*_CTX-M27_ and *bla*_DHA-1_, were also identified. Over 37% of the DEC strains exhibited positivity for one or more ESBL genes. AMR genes for sulphonamide and trimethoprim were detected in 49.5% and 42.9% of the strains, respectively, with 39.6% of the strains exhibiting positivity for both genes. In contrast, AMR genes for phenicol (11.0%), macrolide (30.8%) and tetracycline (27.4%) were identified with comparatively lower frequencies. The non-DEC strains exhibited a similar prevalence of each AMR gene compared to the DEC strains.

Moreover, mutations within the QRDRs of the DEC strains were analysed (Fig. 5 and Table S1). Four, five and two known mutations or combinations thereof in *gyrA*, *parC* and *parE*, respectively, were detected in the strains, with the S83L mutation in *gyrA* (59.3%), including its combination with the D87N mutation, emerging as the most prevalent mutation. At least one known QRDR mutation was identified in 74.7% of DEC strains (84.3% in EAEC, 56.0% in EPEC and 84.3% in ETEC). The mutation rate in the QRDR of non-DEC strains was 35%, approximately half that observed in DEC strains.

**Fig. 6.**
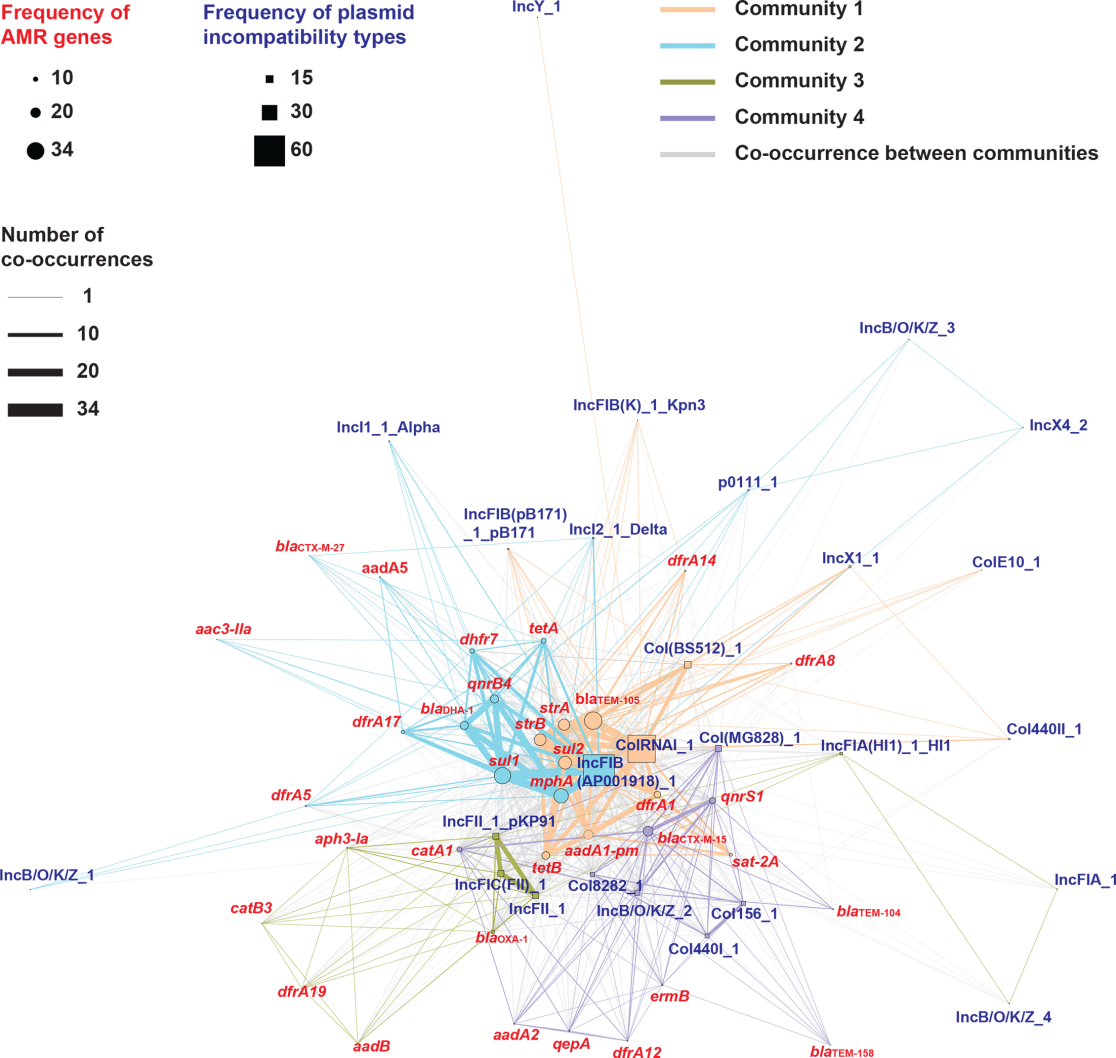
Co-occurrence network analysis of 35 AMR genes and 26 plasmid replicons identified in 91 DEC isolates. The network diagram was edge-weighted according to the frequency of gene co-occurrences, with edge widths representing co-occurrence strength and node sizes reflecting the detection frequency of each gene. The four communities identified are indicated by differently coloured edges.

Subsequently, we conducted plasmid replicon analysis to elucidate the plasmids involved in the dissemination of acquired AMR genes. This analysis revealed 26 distinct plasmid replicon sequences (Table S1). These replicons were heterogeneously distributed across the Indian DEC phylogeny (Fig. 1). Co-occurrence network analysis between plasmid replicon and AMR genes delineated four gene communities (Fig. 6). Frequently identified AMR genes including *bla*_TEM-105_, *strA*, *strB*, *mphA*, *sul1*, *sul2*, *addA1-pm*, *tetA* and *tetB* were clustered into communities 1 and 2, which also encompassed the plasmid replicons ColRNA_1 and IncFIB(AP001918)_1, respectively. These findings suggest that plasmids harbouring ColRNA_1 and IncFIB(AP001918)_1 replicons play a predominant role in the propagation of AMR genes in India.

## Discussion

In this study, to elucidate the genetic features of the major DECs in India, namely, ETEC, EAEC and EPEC [3,5, 6], we analysed the phylogenetic characteristics and distribution of virulence and AMR genes in Indian DEC isolates based on genomic data. The ETEC, EAEC and EPEC strains from India were primarily classified into phylogroups A and B1 (Table 2) as previously reported [36,40]. EPEC and EAEC strains are also frequently classified into phylogroups B2 and D, respectively [36,37, 41, 42]. Consistent with these studies, 28% of the EPEC strains and 25% of the EAEC strains analysed in the present study were assigned to phylogroups B2 and D, respectively. Conversely, a previous study revealed that EAEC strains (*n*=138) derived from children with diarrhoea in northern India were predominantly classified into phylogroup D (43.3%), followed by B1 (24.6%), B2 (23.1%) and A (8.6%) [43]. Potential explanations for this discrepancy include regional variations or temporal differences in sample collection, but this hypothesis needs validation through larger-scale analyses.

Our core gene-based phylogenetic analysis showed that the DEC strains were distributed across various sub-lineages of phylogroups A and B1, irrespective of the pathotype (Fig. 1). Furthermore, although the serotypes were pathotype-specific, several STs included strains of multiple pathotypes. Considering the global isolation of phylogenetically diverse ETEC strains [40], it is postulated that DEC strains are continually emerging through the horizontal transfer of virulence genes, with their transmission occurring globally, including in India [44,48]. Amongst the various STs identified in this study, ST10 and ST443 were predominant and included three different pathotypes (Fig. 1). ST10 is a predominant ST amongst DEC isolates in various other countries, including Nigeria [49], Vietnam [41], Spain [50] and Egypt [51]. Conversely, although an ESBL-producing strain belonging to ST443 was isolated from the bloodstream in Congo [52], there are no reports of ST443 being a predominant ST of DECs. Although larger-scale analyses are needed for validation, these data suggest that ST443 is an India-adapted ST of DECs.

Based on comparative genomics and functional characterization of a collection of EAEC strains in the Global Enteric Multicentre Study (GEMS) [53], Boisen *et al*. proposed defining EAEC as an *E. coli* strain harbouring *aggR* and a complete AAF(I-V) or CS22 gene cluster [36]. In this study, we analysed *aatA-* and/or *aaiC-*positive strains as EAEC. Amongst the 32 EAEC strains analysed, 20 strains were *aggR*-positive, with AAF-I (*n*=4), AAF-II (*n*=1), AAF-III (*n*=4), AAF-V (*n*=7) and CS22 (*n*=2) detected (Table 1). The accessory virulence genes *aap* (dispersin) and *aar* (AggR-activated regulator) and *aatA* were conserved in all the *aggR*-positive EAEC strains (Table S1). Similar to *aggR*, these genes are encoded by the adherence plasmid pAA and are activated by AggR [36,54,56]. The products of these genes play a crucial role in modulating the adherence of EAEC. In the present study, 12 atypical EAEC strains (*aggR*-negative) were isolated from the stool of patients exhibiting diarrhoea. Whilst other causative agents of diarrhoea were detected in some cases, atypical EAEC was identified as the sole causative agent in certain instances (Table S1). Atypical EAEC are frequently isolated from children with diarrhoea, and outbreaks associated with atypical EAEC have been reported [7]. Further investigation is required to elucidate the pathogenic mechanisms underlying atypical EAEC.

Amongst the Indian ETEC strains, the most prevalent toxin type was STa alone (62.5%), followed by the combination of STa and LT1 (34.4%) (Table 1). Only one LT1 single-positive strain was identified. Generally, approximately one-third of ETEC strains isolated from diarrhoeic patients express only LT or only ST, whilst another one-third express both toxin types [7,53]. These data suggest that India is considered an environment conducive to the transmission of ST-positive ETEC strains. As various combinations of toxins and CFs were identified in the ETEC strains in the GEMS [53], the combinations of toxins and CFs were similarly divergent amongst the Indian ETEC strains in this study (Table S1). Various other virulence genes such as *eatA* (serine protease autotransporter), *tia* (invasion determinant) and *east1* (EAEC heat-stable enterotoxin 1) have been identified in ETEC strains [7]. In the present study, *sepA* (serine_protease, 62.5%)*, east1* (56.3%) and *etpA* (two-partner secreted adhesin, 37.5%) were frequently identified in the ETEC strains (Fig. 1 and Table S1). These virulence factors were conserved to a limited extent in non-DEC isolates but may enhance the transmission of ST-positive ETEC strains in India.

Although the *bfpA* gene, a marker for tEPEC, was identified in only two strains, 20 distinct accessory virulence genes were identified in various EPEC strains, exhibiting a range of 0–11 genes with diverse combinations (Fig. 1 and Table S1). These accessory virulence genes are believed to enhance the virulence of EPEC strains [15,16]. A total of 33 non-LEE effectors were identified in the Indian EPEC strains, and their effector repertoires were highly variable amongst the strains (Fig. 3 and Table S1). Although not essential for A/E lesion formation, these non-LEE effectors are thought to increase bacterial virulence [9]. Thirteen different subtypes of intimin were identified, with subtype *eaeA*_β1 (*n*=7) being the most prevalent (Table S1). *eaeA*_β1 has emerged as the most frequent subtype amongst *eaeA*-positive *E. coli* strains globally [57,59]. Interestingly, two ST29 strains (O26:H11 and O109:H49) were found to harbour STEC plasmid- and genomic island-encoded virulence genes, including *ehxA*, *ecf1*, *katP*, *lpxR* and *ureC*. ST29 O26 has been reported as a highly virulent STEC clone that has recently emerged in Europe [60]. These two ST29 aEPEC strains may represent STEC lineages that have lost the *stx* gene. These findings indicate that Indian EPEC strains exhibit substantial genetic diversity, particularly in virulence genes, contributing to their heterogeneous pathogenic potential. Unfortunately, although the DEC strains were isolated from patients with diarrhoea, the lack of detailed symptom data limits our ability to assess the link between virulence traits and clinical manifestations. This issue needs to be clarified in future prospective studies.

The DEC strains analysed in this study included one strain of the EPEC/ETEC pathotype and one strain of the ETEC/EAEC-hybrid pathotype. Infections caused by hybrid pathotypes have sometimes been reported and have been the cause of severe outbreaks, such as the 2011 German outbreak caused by a STEC/EAEC-hybrid pathogen [61]. However, there is insufficient evidence to confirm whether hybrid pathotypes are always more virulent than their parental pathotypes. More information, such as host symptomatology, is necessary to better understand their significance. Additionally, in this study, cryptic clade 1, which carried both *esta* and *elt1*, was isolated from a patient with diarrhoea (Tables 2 and S1). The pathogenicity of cryptic clade 1 is not well understood; however, we previously isolated an STEC/ETEC-hybrid cryptic clade 1 strain from a patient with bloody diarrhoea, and database analysis revealed that many cryptic clade 1 strains possess Stx and/or ETEC enterotoxins [25]. Continuous surveillance of clade 1 infections may be of significant importance for the future control of infections caused by this pathogen.

One to twelve AMR genes were identified in 73.6% of the DEC strains, and 87.5% of the EAEC strains were positive for at least one AMR gene (Fig. 4 and Table S1). Although antibiotic susceptibility testing was not performed in this study, in *E. coli*, *in vitro* AMR phenotypes are largely explained by the presence of known genetic determinants of AMR [62]. Consistent with our study, previous research conducted in India has demonstrated that all EAEC strains exhibited phenotypic resistance to at least one of the tested antimicrobial agents [43]. Additionally, other studies have shown that EAEC strains possess higher resistance frequencies to most tested antimicrobial agents than do ETEC and EPEC strains in India [5]. Considering our findings alongside previous reports, the ecological niche occupied by EAEC in India appears to be more heavily exposed to antimicrobials compared to that of ETEC and EPEC.

The identification of plasmid replicons and their co-occurrence network analysis with AMR genes indicated that plasmids harbouring ColRNA_1 and IncFIB(AP001918)_1 replicons represent key vectors in the propagation of AMR in India (Fig. 1). ColRNA_1 has been characterized as a non-conjugative plasmid encoding the quinolone resistance gene qnrS1 in *Salmonella* spp. [63,64]. IncFIB(AP001918)_1 is frequently detected in *E. coli* strains and has been consistently linked to resistance against a broad spectrum of antimicrobials, including *β*-lactams, aminoglycosides, sulphonamides and tetracyclines [65,67]. A comprehensive analysis of plasmids from Indian DEC isolates using long-read sequencing technologies is imperative to unravel the underlying mechanisms driving the transmission of AMR strains in India.

Nearly 70% of the DEC strains harboured *β*-lactamase genes, and over 37% were positive for ESBL genes (Fig. 5). Consistently, ~80% of DEC strains are reportedly resistant to ampicillin, and third-generation cephalosporin-resistant DEC strains are frequently identified in India [5,43, 68]. Many studies report varying prevalence rates of ESBL-producing DEC in Asia, with the highest at 90.2% in Bangladesh and the lowest at 9.7% in Vietnam [69]. The overall prevalence of ESBL-producing DEC in Asia was estimated to be 48.6% [69]. In the present study, we revealed that acquired quinolone resistance genes and known QRDR mutations were identified in 31.9% and 74.7% of the strains, respectively. Mutations in the QRDR are pivotal in the development of quinolone resistance [70]. Consistent with this study, ~60% of DEC strains in India are resistant to ciprofloxacin and ceftriaxone [5,43, 68]. Ingle *et al*. reported a greater frequency of ciprofloxacin treatment for patients with diarrhoea and the detection of QRDR mutations in DEC strains in India than in other South Asian and sub-Saharan African countries [62]. In Vietnam, in addition to high levels of resistance to *β*-lactams and fluoroquinolones, macrolide resistance genes have been detected in more than 90% of DEC strains [41]. In contrast, strains positive for macrolide resistance genes constituted 27% of the Indian DEC strains (Fig. 5). The similarity in AMR gene acquisition patterns between non-DEC and DEC stool isolates suggests that *E. coli* strains are frequently exposed to corresponding antibiotics in the human population in India (Fig. 5). Our research demonstrates the necessity of continuing localized surveillance of AMR trends in DEC strains and carefully evaluating the choice of antibiotics for diarrhoea treatment.

## Conclusion

Strains of Indian DECs were predominantly classified into either the phylogroup A or B1. They were heterogeneously distributed amongst a wide array of sub-lineages within these phylogroups, lacking a pathotype-specific phylogenetic distribution. This suggests that DECs repeatedly emerge through horizontal gene transfer of hallmark virulence genes, followed by their global spread. The distribution of virulence factors was remarkably diverse, indicating variations in pathogenicity levels amongst strains. The occurrence of human infections caused by emerging pathogens, including hybrid pathotypes and cryptic clade 1, was also identified in India. In line with previous findings, genetic markers indicative of significant AMR, particularly against *β*-lactams and quinolones, were identified in Indian DEC strains. Continuous monitoring of trends in the characteristics of DEC strains is essential for the effective control and treatment of DEC infections in India.

## Supplementary material

10.1099/mgen.0.001430 Uncited Fig. S1.

10.1099/mgen.0.001430 Uncited Table S1.

10.1099/mgen.0.001430 Uncited Table S2.

10.1099/mgen.0.001430 Uncited Table S3.

## Abbreviations

AAF: aggregative adherence fimbriae
A/E: attaching and effacing
aEPEC: atypical EPEC
AMR: antimicrobial resistance
BFP: bundle-forming pilus
CFs: colonization factors
DEC: diarrhoeagenic *Escherichia coli*
EAEC: enteroaggregative *Escherichia coli*
EPEC: enteropathogenic *Escherichia coli*
ESBL: expanded-spectrum *β*-lactamase
ETEC: enterotoxigenic *Escherichia coli*
GEMS: Global Enteric Multicentre Study
LEE: locus of enterocyte effacement
LT: heat-labile enterotoxin
ML: maximum likelihood
QRDRs: quinolone resistance-determining regions
ST: sequence type
ST: heat-stable enterotoxin
STEC: Shiga toxin-producing *Escherichia coli*
Stx: Shiga toxin
tEPEC: typical EPEC
