# Supplementary material for: Genomic features of three major diarrhoeagenic Escherichia coli pathotypes in India
Source: Microb Genom. 2025 Jul 7;11(7):001430. doi: 10.1099/mgen.0.001430 (PMC12282231; doi:10.1099/mgen.0.001430)
Supplement: Uncited Fig. S1. [file mgen-11-01430-s001.pdf]

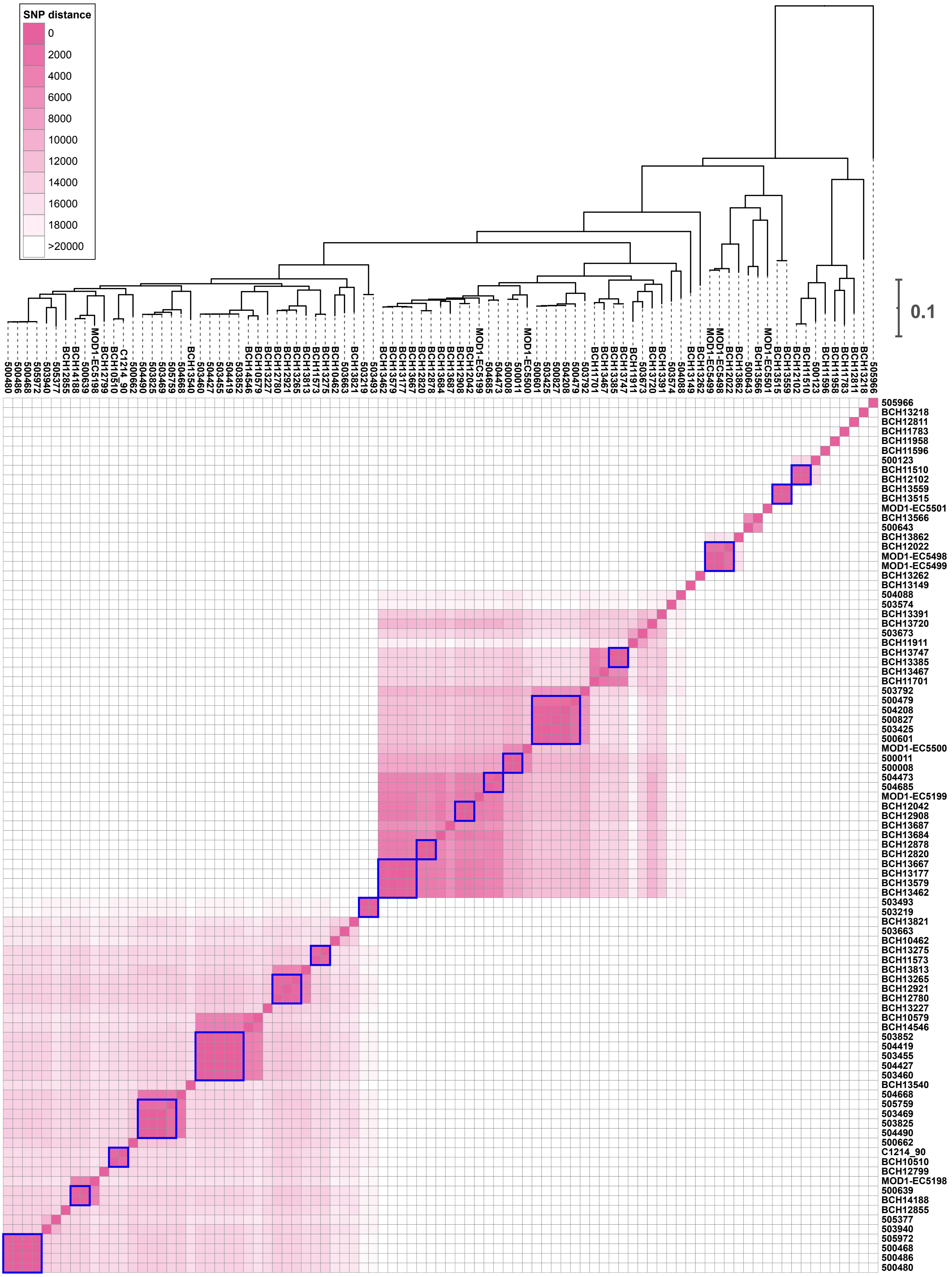

Supplemental figure 1. Heatmap depicting the SNP distances among the 91 isolates analyzed

The phylogenetic tree is consistent with that in Fig. 1. Strains with SNP distances of less than 2000, indicating close relatedness, are highlighted within blue boxes. The color code is provided in the legend above.
